# A Robust Bilinear Framework for Real-Time Speech Separation and Dereverberation in Wearable Augmented Reality

**DOI:** 10.3390/s25175484

**Published:** 2025-09-03

**Authors:** Alon Nemirovsky, Gal Itzhak, Israel Cohen

**Affiliations:** Andrew and Erna Viterbi Faculty of Electrical & Computer Engineering, Technion–Israel Institute of Technology, Haifa 3200003, Israel; alon.nem@campus.technion.ac.il (A.N.); galitz@technion.ac.il (G.I.)

**Keywords:** adaptive beamforming, dereverberation, source separation, region-of-interest beamforming, Kronecker product filtering, augmented reality

## Abstract

This paper presents a bilinear framework for real-time speech source separation and dereverberation tailored to wearable augmented reality devices operating in dynamic acoustic environments. Using the Speech Enhancement for Augmented Reality (SPEAR) Challenge dataset, we perform extensive validation with real-world recordings and review key algorithmic parameters, including the forgetting factor and regularization. To enhance robustness against direction-of-arrival (DOA) estimation errors caused by head movements and localization uncertainty, we propose a region-of-interest (ROI) beamformer that replaces conventional point-source steering. Additionally, we introduce a multi-constraint beamforming design capable of simultaneously preserving multiple sources or suppressing known undesired sources. Experimental results demonstrate that ROI-based steering significantly improves robustness to localization errors while maintaining effective noise and reverberation suppression. However, this comes at the cost of increased high-frequency leakage from both desired and undesired sources. The multi-constraint formulation further enhances source separation with a modest trade-off in noise reduction. The proposed integration of ROI and LCMP within the low-complexity frameworks, validated comprehensively on the SPEAR dataset, offers a practical and efficient solution for real-time audio enhancement in wearable augmented reality systems.

## 1. Introduction

Real-time speech source separation, dereverberation, and background noise suppression are essential to improve intelligibility and audio quality in diverse acoustic environments [1,2,3,4,5]. These processes are vital for applications including augmented reality (AR) [6], teleconferencing, and hearing aids [7,8,9,10,11,12,13]. The increasing demand for robust solutions in dynamic acoustic environments presents considerable challenges, including rapidly changing acoustic conditions, frequent head movements, and the presence of multiple speakers.

Deep learning (DL) methods have transformed the speech enhancement landscape, achieving state-of-the-art results across various tasks, including denoising, dereverberation, and source separation [14]. Architectures such as deep neural networks, recurrent neural networks, convolutional networks, and transformers have demonstrated remarkable effectiveness [15,16,17]. Particularly in challenging dynamic scenarios involving multiple speakers, head movements, and background noise—such as those presented in the SPEAR Challenge—DL models frequently dominate benchmarks [18]. Approaches that combine neural networks with classical beamforming, such as mask-based beamforming [19,20] and fully neural beamformers [21], have been particularly successful.

Despite these advances, DL methods typically require extensive computational resources and large training datasets, limiting their suitability for real-time deployment on wearable AR devices with constrained power and processing capacity. Moreover, their performance often deteriorates in unseen acoustic environments. Consequently, traditional signal processing techniques remain essential in such scenarios.

Beamforming is a spatial filtering technique that combines signals from multiple microphones to emphasize sounds from a desired direction while suppressing sounds from other directions [22,23]. Among the different approaches, the minimum variance distortionless response (MVDR) beamformer [24] is effective at separating sources; however, it often depends on additional information, such as the covariance matrices of the desired source and interference, as well as a voice activity detector (VAD). This reliance on such data can make it challenging to implement MVDR in real-time applications. On the other hand, the minimum power distortionless response (MPDR) beamformer does not require VAD, yet it remains vulnerable to steering vector errors [25], which are inevitable in dynamic environments. Region-of-interest (ROI) beamforming was recently proposed [26,27,28,29,30] to address this issue, offering a near-distortionless response over an angular sector rather than a single direction distortionless response.

Although beamformers suppress directional interference, they do not directly address late reverberation, which can significantly degrade speech intelligibility. Multichannel linear prediction (MCLP) has become a widely adopted method for estimating and removing these reverberant components from observed signals [2]. MCLP can be implemented in both the time domain and the short-time Fourier transform (STFT) domain [31]. Among its variants, the weighted prediction error (WPE) algorithm has shown powerful performance in reducing late reverberation [32,33]. However, additive noise, which exists in realistic acoustic environments, affects the correlation between observations and degrades the dereverberation performance. To mitigate this, beamforming is often applied as a spatial pre-filter to suppress noise and improve the reliability of MCLP-based estimation.

To jointly perform denoising and dereverberation, MCLP-based methods have been integrated with beamforming techniques such as the generalized sidelobe canceller [34], MVDR and weighted MPDR (wMPDR) [35,36]. Although these methods enhance overall performance, they also increase computational complexity. A more efficient approach combines beamforming and dereverberation in a bilinear framework via the Kronecker product [37,38,39]. This approach decouples spatial and temporal filtering, reducing computation while preserving effectiveness. Recently, such a bilinear MPDR-WPE design employing point-source steering with recursive least squares (RLSs) [40,41] cost functions demonstrated promising real-time source separation performance [42].

In this work, we first validate the framework by conducting a comprehensive parameter study of the previously proposed bilinear MPDR-WPE algorithm, assessing the influence of forgetting factor and regularization on real recordings from the SPEAR Challenge dataset [18,43], which captures realistic head-motion and multi-talker AR scenarios. As part of this study, we provide a comprehensive validation of the SPEAR dataset, highlighting its utility and the challenges it presents for robust real-time audio processing. Based on the optimal configurations identified in this study, we then introduce two extensions within the bilinear framework. First, we integrate ROI beamforming to improve resilience to DOA mismatches and reduce sensitivity to head motion. Second, we incorporate a linearly constrained minimum power (LCMP) beamforming design [44,45], enabling greater control over the spatial response—such as preserving multiple sources or suppressing known undesired sources—when additional DOA is available. Experimental results indicate that the baseline MPDR-WPE, its ROI-based variant, and the LCMP extension all effectively reduce interference, mitigate reverberation, and preserve target speech. While ROI steering enhances robustness to localization errors at the cost of some high-frequency leakage, LCMP offers better source separation at the cost of slightly degrading noise reduction. Together, these complementary designs extend the applicability of the framework to a broader range of dynamic wearable AR scenarios.

The remainder of the paper is organized as follows: Section 2 describes the signal model. Section 3 introduces the bilinear framework that combines beamforming with dereverberation. Section 4 presents a real-time RLS-based algorithm tailored for dynamic environments. Section 5 provides a comprehensive dataset and parameter analysis, evaluates ROI beamforming under steering errors, examines associated trade-offs, introduces multi-constraint beamforming, and compares the framework to DL methods.

## 2. Signal Model

We model a dynamic acoustic environment using real recordings from the SPEAR Challenge dataset. The scene includes multiple speakers, reverberation, background noise, and head movements, all captured by an array of *M* microphones. The analysis is performed in the STFT domain.

The multichannel observation vector at time frame *ℓ* and frequency bin *k* is given by

(1) $$y\left(\ell ,k\right)=\left[\begin{array}{c} Y_{1}\left(\ell ,k\right)\\ \vdots \\ Y_{M}\left(\ell ,k\right) \end{array}\right],$$

where $Y_{m}\left(\ell ,k\right)$ denotes the STFT coefficient of the *m*-th microphone. The observed signal can be modeled as

(2) $$y\left(\ell ,k\right)=d\left[\psi (\ell ),k\right]\;X\left(\ell ,k\right)+r\left(\ell ,k\right)+\gamma \left(\ell ,k\right)+\sum_{i}m^{\left(i\right)}\left(\ell ,k\right),$$

where X(ℓ,k) is the desired speech component at the reference microphone, r(ℓ,k) is the late reverberation, γ(ℓ,k) is background noise, and $\sum_{i}m^{\left(i\right)}\left(\ell ,k\right)$ represents interference from speech sources. The vector $d\left[\psi (\ell ),k\right]$ denotes the relative transfer function (RTF) for the target source arriving from direction ψ(ℓ) at time frame *ℓ* and represented by

ψ(ℓ)=[θ(ℓ),ϕ(ℓ)],

where θ(ℓ) is the elevation angle and ϕ(ℓ) is the azimuth angle.

The RTF is defined as

(3) $$d\left[\psi (\ell ),k\right]=\left[\begin{array}{c} \frac{H_{1}\left[\psi (\ell ),k\right]}{H_{\mathrm{ref}}\left[\psi (\ell ),k\right]}\\ \vdots \\ \frac{H_{M}\left[\psi (\ell ),k\right]}{H_{\mathrm{ref}}\left[\psi (\ell ),k\right]} \end{array}\right],$$

where $H_{m}\left[\psi (\ell ),k\right]$ is the acoustic transfer function from the target to the *m*-th microphone, and $H_{\mathrm{ref}}\left[\psi (\ell ),k\right]$ is the transfer function to the chosen reference microphone.

The objective is to estimate X(ℓ,k), the clean target speech at the reference microphone, by suppressing reverberation, background noise, and directional interference in the observed signal y(ℓ,k). For clarity, the frequency index *k* is omitted in the following sections.

## 3. Beamforming and Dereverberation Bilinear Framework

In this section, we extend the bilinear MPDR-WPE framework [42] by introducing two enhancements: ROI beamforming, which improves robustness to DOA errors, and LCMP beamforming, which enables directional interference suppression or multi-target support.

**Figure 1:**
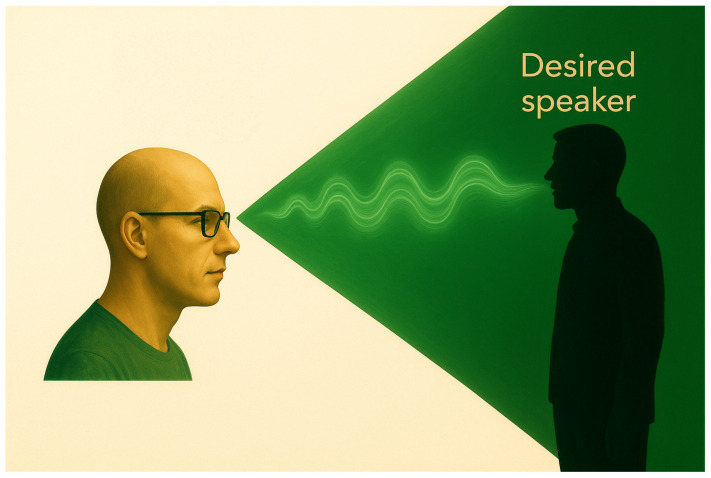
Illustration of the ROI.

The ROI beamformer replaces the traditional point-source distortionless constraint with a least-distortion criterion defined over an angular sector. This formulation reduces sensitivity to steering inaccuracies caused by head movements or localization errors. Specifically, the ROI constraint is given by [29]

(4) $$\Gamma_{d}\left(\Omega \right)\;h=b\left(\Omega \right)$$

where Ω denotes the ROI as illustrated in Figure 1 and

(5) $$\Gamma_{d}\left(\Omega \right)=\frac{1}{\left|\Omega \right|}{\;\int \int \;}_{(\theta ,\phi )\in \Omega }d\left(\theta ,\phi \right)\;d^{H}\left(\theta ,\phi \right)\;\sin\theta \;d\phi \;d\theta ,$$

(6) $$b\left(\Omega \right)=\frac{1}{\left|\Omega \right|}{\;\int \int \;}_{(\theta ,\phi )\in \Omega }d\left(\theta ,\phi \right)\;\sin\theta \;d\phi \;d\theta ,$$

(7) $$\left|\Omega \right|≜{\;\int \int \;}_{(\theta ,\phi )\in \Omega }\sin\theta \;d\theta \;d\phi ,$$

where ${\left(\cdot \right)}^{H}$ denotes the Hermitian transpose.

We also incorporate an LCMP beamformer to support multiple constraints:

(8) $$C^{H}\left(\ell \right)h=\left[\begin{array}{c} 1\\ \beta \end{array}\right],$$

where $C\left(\ell \right)=\left[d\left[\psi^{\left(1\right)}\left(\ell \right)\right]\;d\left[\psi^{\left(2\right)}\left(\ell \right)\right]\right]$ contains the RTFs for two known directions. Setting β=1 preserves both sources (multi-target), while using a small value for β suppresses the second source.

Since the temporal filter is sensitive to additive noise, we first apply the spatial filter h to the multichannel observation y(ℓ):

(9) $$\begin{array}{cc} Z(\ell )\; & =h^{H}\;y\left(\ell \right)\\ \; & =h^{H}\left[d\left[\psi \left(\ell \right)\right]\;X\left(\ell \right)+r\left(\ell \right)+\gamma \left(\ell \right)+\sum_{i}m^{\left(i\right)}\left(\ell \right)\right]\\ \; & =\tilde{X}\left(\ell \right)+\tilde{r}\left(\ell \right)+h^{H}\;v\left(\ell \right), \end{array}$$

where $\tilde{X}\left(\ell \right)$ is the estimated desired speaker (consisting of the direct path and early reverberation) at the reference microphone, which may be slightly distorted due to the relaxed constraint of ROI beamforming, $\tilde{r}\left(\ell \right)$ is the late reverberation component that remains after beamforming and originates from the target speaker, and v(ℓ) is a linear combination of background noise and interference sources.

Next, a temporal filter g of length *L* is applied to suppress the late reverberation that occurs after beamforming. We subtract a predicted reverberant component from Z(ℓ) by applying g to a delayed version of Z(ℓ):

(10) $$\hat{X}\left(\ell \right)=Z\left(\ell \right)-g^{H}\;z\left(\ell -\Delta \right),$$

where Δ is a prediction delay chosen to preserve the correlation of the direct path speech signal. The vector z(ℓ−Δ) contains the previous *L* beamformer outputs and is defined as

(11) $$z\left(\ell -\Delta \right)=\left[\begin{array}{c} Z(\ell -\Delta )\\ Z(\ell -\Delta -1)\\ \vdots \\ Z(\ell -\Delta -L+1) \end{array}\right].$$

Given that h is fixed, the second term in (10) is linear with respect to $g^{H}$, or vice versa. We utilize the Kronecker product representation to estimate the desired signal:

(12) $$\hat{X}\left(\ell \right)=Z\left(\ell \right)-{\left(g⊗h\right)}^{H}\bar{y}\left(\ell -\Delta \right),$$

where ⊗ represents the Kronecker product and $\bar{y}\left(\ell -\Delta \right)$ is the column stacked observation vector of dimension ML:

(13) $$\bar{y}\left(\ell -\Delta \right)=\left[\begin{array}{c} y(\ell -\Delta )\\ y(\ell -\Delta -1)\\ \vdots \\ y(\ell -\Delta -L+1) \end{array}\right].$$

By leveraging the relationship [46]

(14) $$g⊗h=\left(I_{L}⊗h\right)g=\left(g⊗I_{M}\right)h,$$

the spatial and temporal filters can be separated, enabling efficient adaptation in real-time scenarios, with I denoting an identity matrix of appropriate dimension:

(15) $$\begin{array}{cc} \hat{X}\left(\ell \right) & =Z_{h}\left(\ell \right)-g^{H}{\left(I_{L}⊗h\right)}^{H}\bar{y}\left(\ell -\Delta \right)\\ \; & =Z_{h}\left(\ell \right)-g^{H}{\bar{y}}_{h}\left(\ell -\Delta \right), \end{array}$$

where ${\bar{y}}_{h}\left(\ell -\Delta \right)={\left(I_{L}⊗h\right)}^{H}\bar{y}\left(\ell -\Delta \right)$ denotes the observation vector processed by the spatial filter h, and $Z_{h}\left(\ell \right)$ represents the corresponding beamformer output.

Similarly, (12) can be alternatively expressed as

(16) $$\begin{array}{cc} \hat{X}\left(\ell \right) & =h^{H}y\left(\ell \right)-h^{H}{\left(g⊗I_{M}\right)}^{H}\bar{y}\left(\ell -\Delta \right)\\ \; & =h^{H}{\bar{y}}_{g}\left(\ell -\Delta \right), \end{array}$$

where ${\bar{y}}_{g}\left(\ell -\Delta \right)=y\left(\ell \right)-{\left(g⊗I_{M}\right)}^{H}\bar{y}\left(\ell -\Delta \right)$ is the observation signal vector filtered by g.

## 4. Real-Time RLS-Based Bilinear Framework

In this section, we derive a real-time source separation and dereverberation bilinear framework using the RLS algorithm [40] with the time-varying spatial filter h(ℓ) and the time-varying temporal filter g(ℓ). The cost function for the spatial beamformer is defined as follows:

(17) $$L\left[h(\ell )|g(\ell -1)\right]=\sum_{i=1}^{\ell }\alpha^{\ell -i}{\left|h^{H}\left(\ell \right){\bar{y}}_{g(\ell -1)}\left(i-\Delta \right)\right|}^{2}$$

where α is the forgetting factor and

(18) $${\bar{y}}_{g(\ell -1)}\left(i-\Delta \right)=y\left(i\right)-{\left[g\left(\ell -1\right)⊗I_{M}\right]}^{H}\bar{y}\left(i-\Delta \right).$$

The spatial beamformer is obtained by minimizing the cost function L[h(ℓ)|g(ℓ−1)] under various constraints. Although the least-distortion beamformer is mathematically rigorous, it is less suitable for real-time operation. Therefore, we adopt a simpler approach based on the dynamic controlled distortion constraint [26]:

(19) $$\min_{h(\ell )}L\left[h\left(\ell \right)|g\left(\ell -1\right)\right]\;s.t.\;h^{H}\left(\ell \right)b\left(\Omega \right)=1,$$

whose solution is the minimum power ROI beamformer:

(20) $$h\left(\ell \right)=\frac{R_{g}^{-1}\left(\ell \right)b\left(\Omega \right)}{b^{H}\left(\Omega \right)R_{g}^{-1}\left(\ell \right)b\left(\Omega \right)}.$$

Alternatively, the beamformer can be constrained with multiple linear constraints as follows:

(21) $$\min_{h(\ell )}L\left[h\left(\ell \right)|g\left(\ell -1\right)\right]\;s.t.\;C^{H}\left(\ell \right)h\left(\ell \right)=\left[\begin{array}{c} 1\\ \beta \end{array}\right],$$

yielding the LCMP beamformer:

(22) $$h\left(\ell \right)=\frac{R_{g}^{-1}\left(\ell \right)C\left(\ell \right)}{C^{H}\left(\ell \right)R_{g}^{-1}\left(\ell \right)C\left(\ell \right)}\left[\begin{array}{c} 1\\ \beta \end{array}\right],$$

where

(23) $$\begin{array}{cc} R_{g}\left(\ell \right)\; & =\alpha R_{g}\left(\ell -1\right)+{\bar{y}}_{g(\ell -1)}\left(\ell -\Delta \right){\bar{y}}_{g(\ell -1)}^{H}\left(\ell -\Delta \right) \end{array}$$

represents the covariance matrix of ${\bar{y}}_{g(\ell -1)}\left(\ell -\Delta \right)$.

The temporal filter can be iteratively optimized by defining the following cost function:

(24) $$L\left[g\left(\ell \right)|h\left(\ell -1\right)\right]=\sum_{i=1}^{\ell }\alpha^{\ell -i}\frac{{\left|Z_{h(\ell -1)}\left(i\right)-g^{H}\left(\ell \right){\bar{y}}_{h(\ell -1)}\left(i-\Delta \right)\right|}^{2}}{\lambda (i)},$$

where

(25) $${\bar{y}}_{h(\ell -1)}\left(i-\Delta \right)={\left[I_{L}⊗h\left(\ell -1\right)\right]}^{H}\bar{y}\left(i-\Delta \right),$$

and $\lambda \left(\ell \right)=|\hat{X}{\left(\ell \right)|}^{2}$ is the variance of the a priori estimate of the desired signal. This cost function assigns more weight to weaker parts of the signal, such as late reverberation, and less to dominant components like the direct path, making it well-suited for dereverberation.

The solution for the temporal filter g(ℓ) can be obtained by minimizing the cost function L[g(ℓ)|h(ℓ−1)]:

(26) $$g\left(\ell \right)=R_{h}^{-1}\left(\ell \right)p_{h}\left(\ell \right),$$

where $R_{h}\left(\ell \right)$ denotes the weighted covariance matrix of ${\bar{y}}_{h(\ell -1)}\left(\ell -\Delta \right)$ and $p_{h}\left(\ell \right)$ represents the weighted correlation vector between $Z_{h(\ell -1)}\left(\ell \right)$ and ${\bar{y}}_{h(\ell -1)}\left(\ell -\Delta \right)$:

(27) $$\begin{array}{cc} R_{h}\left(\ell \right)\; & =\alpha R_{h}\left(\ell -1\right)+\frac{{\bar{y}}_{h(\ell -1)}\left(\ell -\Delta \right){\bar{y}}_{h(\ell -1)}^{H}\left(\ell -\Delta \right)}{\lambda (\ell )}, \end{array}$$

(28) $$\begin{array}{cc} p_{h}\left(\ell \right) & =\sum_{i=1}^{\ell }\alpha^{\ell -i}\frac{{\bar{y}}_{h(\ell -1)}\left(i-\Delta \right)Z_{h(\ell -1)}^{*}\left(i\right)}{\lambda (i)}. \end{array}$$

In practice, due to the dynamic environment and localization errors, regularization of $R_{g}\left(\ell \right)$ is needed. This can be achieved as follows:

(29) $$R_{\sigma }\left(\ell \right)=R_{g}\left(\ell \right)+\epsilon I_{M},$$

where $\epsilon I_{M}$ is a diagonal matrix with small values with the same dimension as the sensors.

Computational complexity can be improved using Woodbury’s identity [40]. The updates of $R_{h}^{-1}\left(\ell \right)$, $R_{g}^{-1}\left(\ell \right)$, and $R_{\sigma }^{-1}\left(\ell \right)$ are as follows:

(30) $$R_{h}^{-1}\left(\ell \right)=\frac{I_{L}-k_{h}\left(\ell \right){\bar{y}}_{h(\ell -1)}^{H}\left(\ell -\Delta \right)}{\alpha }R_{h}^{-1}\left(\ell -1\right)$$

(31) $$R_{g}^{-1}\left(\ell \right)=\frac{I_{M}-k_{g}\left(\ell \right){\bar{y}}_{g(\ell -1)}^{H}\left(\ell -\Delta \right)}{\alpha }R_{g}^{-1}\left(\ell -1\right)$$

(32) $$R_{\sigma }^{-1}\left(\ell \right)=R_{g}^{-1}\left(\ell \right)-R_{g}^{-1}\left(\ell \right){\left[\epsilon^{-1}I_{M}+R_{g}^{-1}\left(\ell \right)\right]}^{-1}R_{g}^{-1}\left(\ell \right)$$

where

(33) $$\begin{array}{cc} k_{h}\left(\ell \right) & =\frac{R_{h}^{-1}\left(\ell -1\right){\bar{y}}_{h(\ell -1)}\left(\ell -\Delta \right)}{\alpha \lambda \left(\ell \right)+{\bar{y}}_{h(\ell -1)}^{H}\left(\ell -\Delta \right)R_{h}^{-1}\left(\ell -1\right){\bar{y}}_{h(\ell -1)}\left(\ell -\Delta \right)} \end{array}$$

(34) $$\begin{array}{cc} k_{g}\left(\ell \right) & =\frac{R_{g}^{-1}\left(\ell -1\right){\bar{y}}_{g(\ell -1)}\left(\ell -\Delta \right)}{\alpha +{\bar{y}}_{g(\ell -1)}^{H}\left(\ell -\Delta \right)R_{g}^{-1}\left(\ell -1\right){\bar{y}}_{g(\ell -1)}\left(\ell -\Delta \right)} \end{array}$$

are the Kalman gains.

The temporal filter can be iteratively updated by the derived Kalman gain:

(35) $$g\left(\ell \right)=g\left(\ell -1\right)+k_{h}\left(\ell \right){\hat{X}}^{*}\left(\ell \right).$$

As shown in [37], this approach significantly reduces the computational complexity from $O(M^{2}L^{2})$ to $O(M^{2}+L^{2})$ by combining two sub-optimal solutions via the Kronecker product.

## 5. Experimental Results

**Figure 2:**
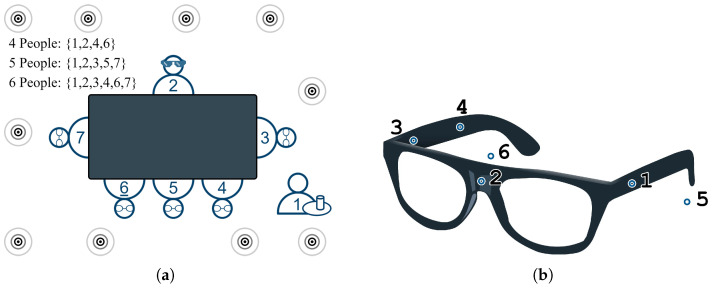
(**a**) SPEAR Challenge scene setup showing speaker positions around the table. (**b**) Head-mounted microphone array worn by person ID2, with six microphones labeled 1–6 [18,43].

In this section, we present experimental results using the first dataset from the SPEAR Challenge. This dataset provides real six-channel recordings captured with a head-worn microphone array in a highly dynamic, noisy, and reverberant environment (Figure 2). The recording room measures 6.11×7.74×3.44m and has a reverberation time of $T_{60}=645\;\mathrm{ms}$. The dataset includes speech from both a nearby (worn) speaker and distant speakers, alongside continuous head movements by the microphone wearer. Additionally, ten loudspeakers placed at different heights throughout the room emit uncorrelated, restaurant-like background noise. These combined factors—and the use of real, non-simulated recordings—make the task particularly challenging, resulting in relatively large standard deviations across evaluation metrics that reflect the inherent variability of the environment.

To accurately capture the array’s spatial characteristics, the dataset includes 1020 sphere-sampled points with corresponding impulse responses (IRs) measured on a mannequin in an anechoic chamber. We employed the Haversine formula [47] to identify the nearest-neighbor IR for each sampled direction, then derived the RTF with respect to sensor 2, which served as the reference sensor. Inevitably, steering errors emerged due to the finite sampled sphere points, head movements, and reverberation.

Each audio file is one minute long and was originally sampled at 48 kHz. For computational efficiency, we downsampled the recordings to 16 kHz, processed them using 1024-sample STFT frames (with 75% overlap and a Hamming window), and applied a prediction delay of Δ=2. The WPE filter lengths were set to 14, 12, and 10 taps for the 0–1 kHz, 1–3 kHz, and 3–8 kHz bands, respectively. The spatial filter, comprising M=6 microphones, was initially configured as a delay-and-sum beamformer, and the inverse covariance matrices were set to $R_{g}^{-1}\left(0\right)=10^{-2}I_{M}$ and $R_{h}^{-1}\left(0\right)=10^{-4}I_{L}$. We used a forgetting factor α=0.994 and a regularization factor of ϵ=0.01, which together ensured convergence within a few hundred frames while balancing noise suppression and target preservation. Experiments were implemented in MATLAB (R2023b; MathWorks, Natick, MA, USA) and executed on a laptop with an Intel i7-1360P CPU (Intel Corporation, Santa Clara, CA, USA) and 16 GB RAM, demonstrating its feasibility for deployment in AR applications with comparable computational resources.

The source separation task was carried out by designating either Person 6 (ID6) or Person 4 (ID4) as the target speaker in separate experiments. Person 2 (the microphone wearer) and either Person 4 or Person 6, depending on the setup, were treated as interference speakers, with background noise serving as additional interference. Since clean reference signals were unavailable, a pseudo ground truth was generated using an MVDR-VAD beamformer that updates its noise covariance matrix only during intervals when the target speaker is silent, as described in [48], and nulls the signal during these periods. This approach is practical for deriving stable references; however, since MVDR–VAD is itself a beamforming method, it may introduce a bias in favor of beamforming-style algorithms and underestimate the performance of fundamentally different approaches such as deep learning models. All algorithm outputs were evaluated against this pseudo ground truth, and performance metrics, as reported in the tables, were averaged across five distinct one-minute recordings.

For quantitative evaluation, we employed several objective measures. Perceptual evaluation of speech quality (PESQ) [49] and short-time objective intelligibility (STOI) [50] assess overall speech quality and intelligibility, respectively. In addition, we computed the scale-invariant signal-to-distortion ratio (SI-SDR) [51], the segmental signal-to-noise ratio (SNRseg), and the frequency-weighted segmental SNR (fwSegSNR) [52] to quantify desired signal preservation and noise suppression. We also measured the average maximum correlation (AMC) [42], which evaluates the correlation of each speaker in the output relative to the MVDR-VAD reference. Lower AMC indicates stronger suppression of undesired sources, while higher AMC implies better preservation of the target. The results are reported as performance differences (Δ) between the proposed algorithm and the reference microphone, with both compared to the pseudo-ground truth.

In the following subsections, assuming an elevation of 0 degrees, we present a comprehensive parameter analysis, beginning with the forgetting factor and regularization under a point-source model, and proceeding through ROI-based beamforming, azimuthal error sensitivity, multi-constraint beamforming, and concluding with a comparison to DL methods.

### 5.1. Forgetting Factor Analysis

The forgetting factor α in the RLS-based MPDR-WPE algorithm controls the balance between fast adaptation and stable convergence. Lower values of α allow quick tracking of changes but may lead to instability. Higher values improve stability but slow down the adaptation process. We tested three values: α∈{0.99,0.994,0.9994}, under a point-source setup, and compared them with two baseline methods using α=0.994 and ϵ=0.01: MPDR and wMPDR-WPE. Table 1 shows that the MPDR-WPE algorithm with α=0.994 offers the best balance across all metrics. A lower value (α=0.99) caused the algorithm to diverge, while a higher value (α=0.9994) resulted in slower adaptation and degraded performance.

Compared to the baselines, MPDR performs worse overall, as its ability to adapt is limited and it does not explicitly address reverberation. wMPDR-WPE shows strong intelligibility and target preservation but is less effective at suppressing interfering sources. As discussed in [42], this highlights a common trade-off: wMPDR-WPE prioritizes target preservation and background noise reduction, while MPDR-WPE emphasizes directional interference suppression and source separation. Despite introducing some distortion to the desired signal, MPDR-WPE provides better directional noise suppression, making it more suitable for source separation tasks in our setting.

**Figure 3:**
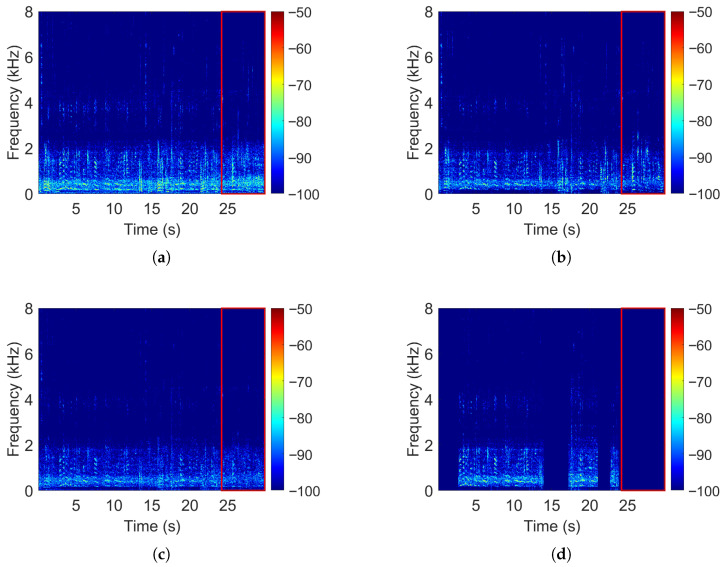
Spectrogram comparisons for (**a**) MPDR, (**b**) wMPDR-WPE, (**c**) MPDR-WPE, and (**d**) MVDR-VAD (ground truth). The red box highlights the interfering speech signal from Person 2 (microphone wearer).

Figure 3 illustrates these differences. The proposed MPDR-WPE algorithm shows the strongest suppression of Person 2’s speech (red box), with better directional interference attenuation than both MPDR and wMPDR-WPE.

In summary, a forgetting factor of α=0.994 provides the most effective balance between adaptation speed and convergence stability for MPDR-WPE. Lower values lead to instability, while higher values slow adaptation and reduce performance. Compared to MPDR and wMPDR-WPE, the proposed method achieves superior source separation while maintaining strong intelligibility and noise suppression. These results make MPDR-WPE with α=0.994 a compelling choice for real-time speech enhancement in dynamic environments.

### 5.2. Regularization

Next, we investigate the effect of different regularization values ϵ on the point-source MPDR-WPE framework [42]. Regularizing the time-varying covariance estimate $R_{g}\left(\ell \right)$ helps maintain robust filtering, particularly due to the MPDR beamformer’s vulnerability to steering errors in dynamic environments. We tested ϵ∈{0,0.01,0.1}, representing no regularization, moderate regularization, and stronger regularization, respectively.

Table 2 shows that ϵ=0 can yield strong performance but risks damaging the desired speaker. A larger ϵ (e.g., 0.1) broadens the beam, effectively preserving the target, but reduces SNR and quality gains. Overall, ϵ=0.01 provides both robustness and high performance. We therefore adopt ϵ=0.01 in subsequent experiments.

### 5.3. Region of Interest Impact

To demonstrate the effect of an ROI-based beamformer, we compared it against a point-source design assuming a precisely known DOA. In the ROI-based approach, the beamformer accommodates localization uncertainty by defining a symmetric angular sector around the nominal direction, which is set to $\Phi_{\mathrm{ROI}}\in \left[\phi_{\mathrm{DOA}}-18^{\circ },\phi_{\mathrm{DOA}}+18^{\circ }\right]$.

**Figure 4:**
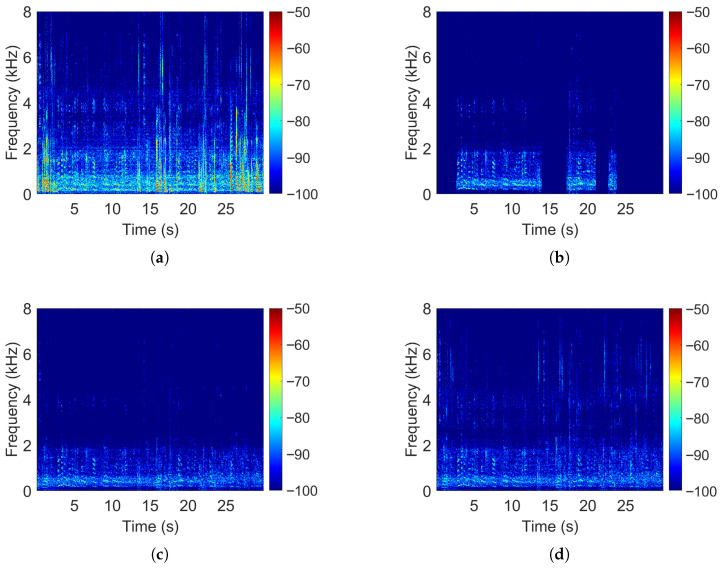
Spectrogram comparisons for (**a**) the reference microphone, (**b**) MVDR-VAD (ground truth), (**c**) MPDR-WPE, and (**d**) an ROI-based MPDR-WPE.

As shown in Figure 4, both beamformers produce similar outputs below about 3 kHz. Above that frequency, however, the ROI-based beamformer passes more high-frequency energy from both the desired and interfering sources. We interpret this as a form of “high-frequency regularization.” At lower frequencies, small changes in steering direction result in negligible variations in the steering vectors, which explains the minor differences between the ROI and point-source beamformers in this range. In contrast, at higher frequencies, the steering vectors vary more rapidly, so the ROI-based beamformer preserves more energy from those bands.

**Figure 5:**
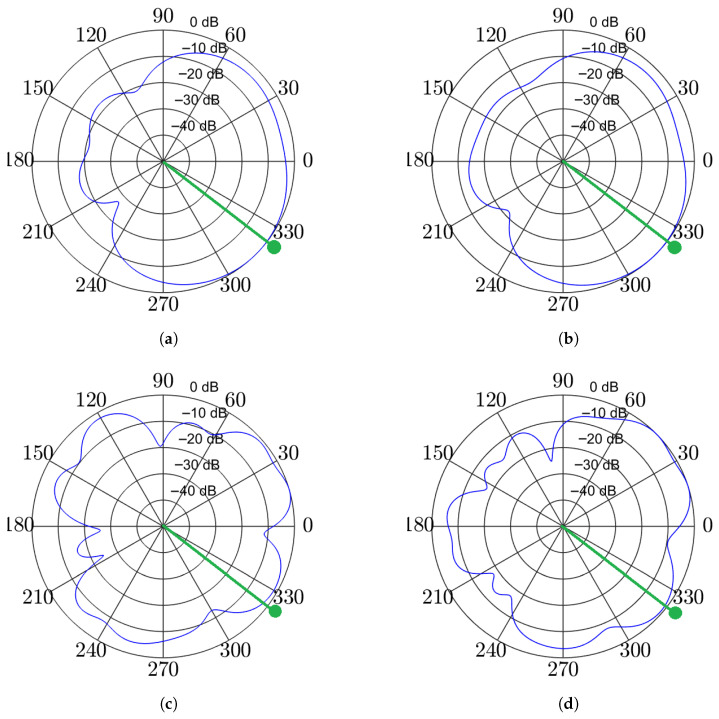
Example beampatterns at 1 kHz (top row) and 4 kHz (bottom row). The beamformer is steered toward a speaker at approximately $327^{\circ }$ (green line with dot). The blue lines represent the resulting spatial beampatterns. Figures (**a**,**c**) show a point-source MPDR-WPE, while (**b**,**d**) illustrate the ROI-based MPDR-WPE.

Figure 5 further validates our thesis. At 1 kHz, both ROI-based and point-source beamformers yield similar beampatterns. In contrast, at 4 kHz, the differences are more pronounced, especially near the target direction. The ROI-based design broadens the pass region, providing more robust preservation of the target speech in dynamic environments, which inevitably involve steering errors. This advantage comes at the cost of passing more interference from nearby angles. In general, ROI-based beamforming offers a practical compromise between strong performance and resilience to moderate angular mismatches.

### 5.4. Azimuthal Error Sensitivity Analysis

**Figure 6:**
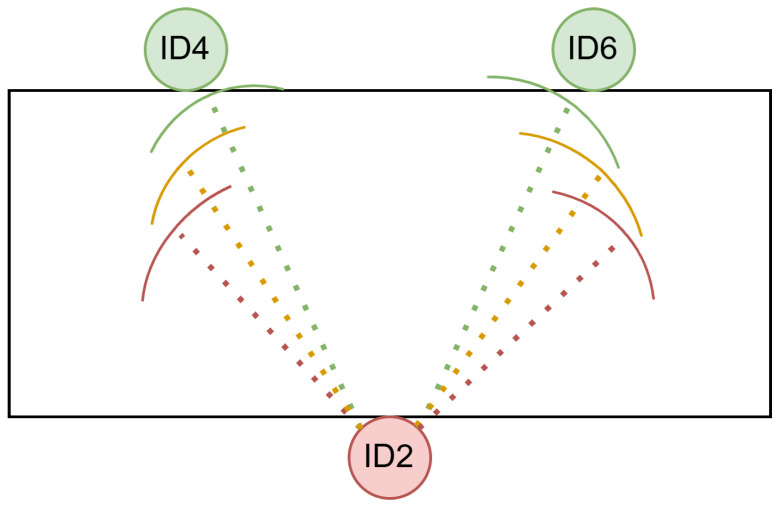
Schematic illustration of the azimuth-error experiment. The microphone wearer (ID2) is located at the bottom, with two potential target speakers (ID4, ID6) in front. The dashed lines indicate possible DOAs under varying steering errors: green (no error), yellow ($12^{\circ }$), and red ($18^{\circ }$).

Next, we examine how azimuthal steering inaccuracies affect the two beamformer designs (point source versus ROI) for speakers ID4 and ID6. Assuming that the given direction is precise, three levels of azimuth error were introduced synthetically ($0^{\circ }$, $12^{\circ }$, and $18^{\circ }$), simulating realistic mismatches between the actual speaker location and the steered beam. The ROI was defined as $\Phi_{\mathrm{ROI}}\in \left[\phi -24^{\circ },\phi +24^{\circ }\right]$, as illustrated in Figure 6.

To better understand the frequency-dependent behavior of ROI beamforming under azimuthal mismatches, we divide the results into full-band and high-frequency (3–8 kHz) evaluations. This distinction is motivated by our earlier observations in Figure 4 and Figure 5, which showed that ROI-based designs diverge most significantly from point-source beamformers above approximately 3 kHz. In this regime, steering vectors change more rapidly with angle, making the ROI constraint more influential. Separating the metrics allows us to evaluate this trade-off more precisely, highlighting how the ROI beamformer improves robustness to angular error at the cost of increased high-frequency leakage.

Table 3 and Table 4 show that when there is no steering error, the point-source design generally performs best. However, as the azimuth error grows, the mismatch between the true target direction and the beam steering leads to significant performance degradation for the point-source beamformer. The ROI-based design proves more resilient to these errors—especially in SI-SDR—by admitting a spatial sector rather than a single direction. This broader pass region better preserves the desired speaker under moderate localization inaccuracies.

At higher frequencies, the ROI beamformer also allows more undesired signals to pass, resulting in reduced SNR gains compared to the strictly point-focused approach. Hence, while the ROI beamformer maintains robust preservation of the desired signal when azimuth errors increase, it comes with the trade-off of higher interference leakage in the upper frequency range.

### 5.5. Multi-Constraint Beamforming

We next explore an extension of the bilinear framework using an LCMP beamformer with multiple directional constraints. In the first setup, the beamformer is steered toward the desired speaker while strongly attenuating the undesired distant speaker by applying a −40dB constraint in that direction. Table 5 compares this LCMP-WPE configuration with the MPDR-WPE framework and a standard LCMP beamformer.

Table 5 shows that LCMP-WPE improves directional interference suppression compared to MPDR-WPE, as indicated by the AMC scores. It also improves signal quality. However, its intelligibility and noise suppression are slightly lower than MPDR-WPE, reflecting the trade-off introduced by the additional constraint. Compared to a simple LCMP beamformer, the LCMP-WPE framework introduces much better results.

We also evaluate LCMP-WPE in a dual-target setup that preserves both ID4 and ID6. In this experiment only, the pseudo ground truth is constructed by combining the individual MVDR-VAD outputs for each desired speaker. The results are shown in Table 6.

As shown in Table 6, LCMP-WPE can preserve multiple speakers while suppressing interference and reverberation. It consistently outperforms LCMP in all metrics. Although the performance per speaker is lower than that of MPDR-WPE in a single-target setup, LCMP-WPE is more suitable when multiple sources must be enhanced simultaneously. For single-speaker scenarios, MPDR-WPE remains the preferred choice.

### 5.6. Comparison with Deep Learning Methods

The proposed bilinear framework and DL approaches, such as hybrid subband-fullband gated convolutional recurrent networks, represent two fundamentally different paradigms for multichannel speech enhancement. DL methods have demonstrated strong performance in benchmark settings, often surpassing traditional techniques in metrics like PESQ and SI-SDR when sufficient in-domain training data and computational resources are available [18,53]. However, they also pose practical limitations, particularly for real-time deployment in wearable AR systems.

From a generalization perspective, DL models typically exhibit sensitivity to mismatches between training and test conditions, including changes in speaker characteristics or room acoustics. This vulnerability arises from their reliance on supervised learning over finite datasets, which may encode bias toward the training domain [54]. In contrast, our bilinear framework method requires minimal prior training and adapts online to dynamic acoustic environments, including head rotations and moving speakers.

In terms of computational demands, DL systems often involve millions of parameters and billions of multiply–accumulate operations per second [55], resulting in high energy consumption and considerable latency. This hinders real-time execution on battery-powered AR devices. Our framework, on the other hand, operates with minimal memory and processing overhead, introducing a theoretical delay of only two frames, making it highly suitable for low-latency applications.

Another key distinction lies in interpretability and control. DL models function as black boxes and may hallucinate non-existent speech components or introduce perceptual artifacts under unseen conditions [56]. In contrast, our bilinear framework is based on well-established signal processing principles, offering predictable behavior and explicit control over spatial filtering and dereverberation. While DL may excel in perceptual metrics under favorable conditions, our method offers a transparent, adaptive, and computationally efficient alternative, providing faithful signal reconstruction that aligns well with the real-time requirements of wearable AR audio systems.

## 6. Conclusions

We have presented a comprehensive evaluation of a bilinear framework for real-time source separation and dereverberation in wearable AR systems, using the SPEAR dataset. We analyzed the influence of key parameters, including the forgetting factor and regularization strength, on the algorithm’s robustness in dynamic acoustic environments. To improve resilience against steering inaccuracies caused by localization uncertainty and head movements, the framework was extended with ROI beamforming, replacing point-source steering with an angular sector constraint. Experimental results demonstrate that ROI-based steering enhances preservation of the desired source under mismatches, particularly at high frequencies, though at the cost of increased off-axis leakage. We further introduced an LCMP-based extension that provides enhanced spatial control by enabling the enforcement of multiple directional constraints. This makes LCMP particularly effective when multiple DOA priors are available, allowing the beamformer to simultaneously preserve or suppress signals from several known directions. While LCMP improves source separation under these conditions, it may introduce a slight compromise in noise suppression.

Compared to deep learning-based approaches, the proposed bilinear framework offers strong real-time adaptability, robustness to unseen environments, and low computational complexity, making it highly suitable for deployment on wearable AR platforms. Future work will explore adaptive ROI strategies that adjust the angular sector in response to localization confidence and investigate the integration of data-driven beamforming with WPE to further enhance performance in challenging acoustic scenes. Another direction is to incorporate perceptual cues beyond spatial information. In particular, pitch-informed beamforming and loudness-based source weighting could improve robustness when spatial cues are degraded by reverberation or head motion.

## Figures and Tables

**Table 1:** Comparison of forgetting factor values (α) for Person 4 (ID4) and Person 6 (ID6). The algorithm does not converge for α=0.99. Bold values indicate the best performance for each metric and each desired speaker.

Metric	MPDR [48]		wMPDR-WPE [37]		α=0.994		α=0.9994	
	ID4	ID6	ID4	ID6	ID4	ID6	ID4	ID6
ΔPESQ	−0.73±1.12	−0.59±1.34	0.61±0.74	0.28±0.74	0.77±1.42	0.16±0.99	0.23±0.53	0.47±0.99
ΔSTOI	0.11±0.02	0.13±0.02	0.22±0.03	0.19±0.04	0.17±0.02	0.16±0.04	0.08±0.02	0.11±0.03
ΔfwSegSNR	0.24±0.09	0.81±0.55	0.82±0.33	1.43±0.75	0.54±0.11	1.44±1.12	0.11±0.07	0.92±1.00
ΔSI-SDR	9.91±3.97	10.28±1.99	10.13±3.23	9.85±2.72	9.29±4.74	9.70±3.42	7.41±3.05	7.85±1.89
ΔSNRseg	0.97±0.39	1.72±0.72	1.83±0.78	2.68±1.20	1.58±0.50	2.72±1.43	0.72±0.32	1.73±1.17
Δ ${\mathrm{AMC}}_{\mathrm{ID}2}$	−0.22±0.06	−0.27±0.03	−0.27±0.06	−0.29±0.03	−0.33±0.06	−0.35±0.05	−0.09±0.05	−0.14±0.03
Δ ${\mathrm{AMC}}_{\mathrm{ID}4}$	0.16±0.03	−0.12±0.04	0.31±0.03	−0.03±0.04	0.27±0.03	−0.12±0.03	0.12±0.03	−0.12±0.04
Δ ${\mathrm{AMC}}_{\mathrm{ID}6}$	−0.13±0.02	0.18±0.04	−0.05±0.05	0.27±0.07	−0.13±0.05	0.25±0.07	−0.11±0.05	0.16±0.05

**Table 2:** Comparison of regularization values (ϵ) for both ID4 and ID6 as the desired source. Bold values indicate the best performance for each metric and each desired speaker.

Metric	ϵ=0		ϵ=0.01		ϵ=0.1	
	ID4	ID6	ID4	ID6	ID4	ID6
ΔPESQ	0.84±1.26	0.27±0.91	0.77±1.42	0.16±0.99	0.77±1.27	−0.09±0.59
ΔSTOI	0.18±0.01	0.16±0.04	0.17±0.02	0.16±0.04	0.14±0.01	0.14±0.03
ΔfwSegSNR	0.61±0.12	1.50±1.15	0.54±0.11	1.44±1.12	0.39±0.10	1.25±1.03
ΔSI-SDR	9.17±4.82	9.49±3.55	9.29±4.74	9.70±3.42	9.33±4.55	9.80±3.08
ΔSNRseg	1.62±0.50	2.72±1.43	1.58±0.50	2.72±1.43	1.41±0.47	2.53±1.35
Δ ${\mathrm{AMC}}_{\mathrm{ID}2}$	−0.33±0.06	−0.35±0.05	−0.33±0.06	−0.35±0.05	−0.29±0.07	−0.32±0.05
Δ ${\mathrm{AMC}}_{\mathrm{ID}4}$	0.29±0.03	−0.12±0.02	0.27±0.03	−0.12±0.03	0.12±0.03	−0.12±0.03
Δ ${\mathrm{AMC}}_{\mathrm{ID}6}$	−0.14±0.05	0.25±0.07	−0.13±0.05	0.25±0.07	−0.12±0.04	0.22±0.06

**Table 3:** Comparison of Point and ROI configurations for full-band and high-frequency (3 kHz–8 kHz) performance across various azimuth errors for person ID4 as the desired speaker. Bold values indicate the best performance for each metric under each error condition.

Metric (ID4)	No Error		12° Error		18° Error	
	Point	ROI	Point	ROI	Point	ROI
**Full-Band**						
ΔPESQ	0.77±1.42	0.64±1.54	0.62±0.84	0.83±1.20	0.67±1.28	0.17±0.89
ΔSTOI	0.17±0.02	0.14±0.02	0.08±0.02	0.09±0.01	0.03±0.02	0.05±0.01
ΔfwSegSNR	0.54±0.11	0.40±0.10	0.27±0.09	0.32±0.09	0.14±0.12	0.24±0.10
ΔSI-SDR	9.29±4.74	9.01±4.64	8.08±4.87	8.40±4.82	6.68±4.77	7.45±4.76
ΔSNRseg	1.58±0.50	1.29±0.41	1.34±0.44	1.23±0.39	1.22±0.41	1.14±0.37
**High-Frequency Section (3 kHz–8 kHz)**						
ΔSTOI	0.21±0.02	0.11±0.02	0.04±0.02	0.07±0.03	0.00±0.03	0.04±0.03
ΔfwSegSNR	0.74±0.41	0.30±0.19	0.24±0.27	0.20±0.12	0.01±0.15	0.14±0.08
ΔSNRseg	1.82±0.76	0.43±0.19	0.97±0.47	0.59±0.25	0.80±0.38	0.62±0.26

**Table 4:** Comparison of Point and ROI configurations for full-band and high-frequency (3 kHz–8 kHz) performance across various azimuth errors for person ID6 as the desired speaker. Bold values indicate the best performance for each metric under each error condition.

Metric (ID6)	No Error		12° Error		18° Error	
	Point	ROI	Point	ROI	Point	ROI
**Full-Band**						
ΔPESQ	0.16±0.99	0.26±0.71	0.38±0.72	0.28±0.84	0.65±1.11	0.31±0.78
ΔSTOI	0.16±0.04	0.14±0.03	0.05±0.03	0.07±0.03	0.00±0.04	0.02±0.04
ΔfwSegSNR	1.44±1.12	0.81±0.43	0.69±0.54	0.63±0.35	0.49±0.39	0.48±0.27
ΔSI-SDR	9.70±3.42	9.12±3.17	8.53±3.76	8.53±3.23	7.20±3.93	7.73±3.30
ΔSNRseg	2.72±1.43	2.10±1.00	1.97±0.89	1.89±0.86	1.79±0.76	1.71±0.75
**High-Frequency Section (3 kHz–8 kHz)**						
ΔSTOI	0.19±0.06	0.08±0.04	−0.01±0.05	0.03±0.04	−0.06±0.06	−0.01±0.05
ΔfwSegSNR	1.62±1.12	0.57±0.28	0.59±0.40	0.53±0.27	0.27±0.25	0.44±0.22
ΔSNRseg	3.24±1.71	0.63±0.34	1.43±0.60	0.85±0.40	1.19±0.48	0.91±0.43

**Table 5:** Performance comparison of LCMP, MPDR-WPE, and LCMP-WPE in a single-target scenario for speakers ID4 and ID6. LCMP and LCMP-WPE apply a −40dB directional constraint toward the undesired speaker. Bold values indicate the best performance for each metric and each speaker.

Metric	LCMP		MPDR-WPE		LCMP-WPE	
	ID4	ID6	ID4	ID6	ID4	ID6
ΔPESQ	0.35±1.64	0.18±1.05	0.77±1.42	0.16±0.99	0.90±0.82	0.67±0.54
ΔSTOI	0.02±0.01	0.01±0.02	0.17±0.02	0.16±0.04	0.11±0.01	0.09±0.01
ΔfwSegSNR	−0.04±0.11	0.24±0.22	0.54±0.11	1.44±1.12	0.37±0.07	0.85±0.45
ΔSI-SDR	4.91±3.01	5.50±1.65	9.29±4.74	9.70±3.42	9.10±4.58	9.39±3.00
ΔSNRseg	0.18±0.08	0.25±0.16	1.58±0.50	2.72±1.43	1.32±0.39	2.00±0.90
Δ ${\mathrm{AMC}}_{\mathrm{ID}2}$	−0.07±0.05	−0.16±0.03	−0.33±0.06	−0.35±0.05	−0.35±0.07	−0.37±0.03
Δ ${\mathrm{AMC}}_{\mathrm{ID}4}$	0.05±0.02	−0.16±0.04	0.27±0.03	−0.12±0.03	0.21±0.04	−0.18±0.04
Δ ${\mathrm{AMC}}_{\mathrm{ID}6}$	−0.19±0.03	0.05±0.03	−0.13±0.05	0.25±0.07	−0.21±0.03	0.19±0.05

**Table 6:** Performance of LCMP and LCMP-WPE in a dual-target configuration, preserving both ID4 and ID6. Bold values indicate the best performance for each metric.

Metric	LCMP	LCMP-WPE
ΔPESQ	−0.07±0.34	0.73±0.55
ΔSTOI	0.02±0.01	0.07±0.01
ΔfwSegSNR	0.10±0.22	0.87±0.47
ΔSI-SDR	2.07±0.95	6.99±3.61
ΔSNRseg	0.35±0.27	2.63±1.14
Δ ${\mathrm{AMC}}_{\mathrm{ID}2}$	0.00±0.01	−0.32±0.06
Δ ${\mathrm{AMC}}_{\mathrm{ID}4}$	0.02±0.02	0.12±0.03
Δ ${\mathrm{AMC}}_{\mathrm{ID}6}$	0.05±0.03	0.14±0.06

## Data Availability

Publicly available datasets were analyzed in this study. These data can be found here: SPEAR Challenge dataset—https://imperialcollegelondon.github.io/spear-challenge/ (accessed on 21 July 2025).

## Abbreviations

The following abbreviations are used in this manuscript:

AMC	Average Maximum Correlation
AR	Augmented Reality
DL	Deep Learning
DOA	Direction of Arrival
fwSegSNR	Frequency-Weighted Segmental SNR
IR	Impulse Response
LCMP	Linearly Constrained Minimum Power
MCLP	Multichannel Linear Prediction
MPDR	Minimum Power Distortionless Response
MVDR	Minimum Variance Distortionless Response
PESQ	Perceptual Evaluation of Speech Quality
RLS	Recursive Least Squares
ROI	Region of Interest
RTF	Relative Transfer Function
SI-SDR	Scale-Invariant Signal-to-Distortion Ratio
SNRseg	Segmental Signal-to-Noise Ratio
SPEAR	Speech Enhancement for Augmented Reality
STFT	Short-Time Fourier Transform
STOI	Short-Time Objective Intelligibility
VAD	Voice Activity Detector
wMPDR	Weighted Minimum Power Distortionless Response
WPE	Weighted Prediction Error

## References

[1] Schmid D., Enzner G., Malik S., Kolossa D., Martin R. (2014). Variational Bayesian Inference for Multichannel Dereverberation and Noise Reduction. IEEE/ACM Trans. Audio Speech Lang. Process..

[2] Nakatani T., Yoshioka T., Kinoshita K., Miyoshi M., Juang B.-H. (2010). Speech Dereverberation Based on Variance-Normalized Delayed Linear Prediction. IEEE Trans. Audio Speech Lang. Process..

[3] Löllmann H.W., Brendel A., Kellermann W. Generalized coherence-based signal enhancement. Proceedings of the 45th IEEE International Conference on Acoustics, Speech and Signal Processing (ICASSP).

[4] Ikeshita R., Kinoshita K., Kamo N., Nakatani T. (2021). Online Speech Dereverberation Using Mixture of Multichannel Linear Prediction Models. IEEE Signal Process. Lett..

[5] Nakatani T., Ikeshita R., Kinoshita K., Sawada H., Araki S. Blind and Neural Network-Guided Convolutional Beamformer for Joint Denoising, Dereverberation, and Source Separation. Proceedings of the 46th IEEE International Conference on Acoustics, Speech and Signal Processing (ICASSP).

[6] Greengard S. (2019). Virtual Reality.

[7] Doclo S., Gannot S., Moonen M., Spriet A. (2010). Acoustic Beamforming for Hearing Aid Applications. Handbook on Array Processing and Sensor Networks.

[8] Westhausen N.L., Kayser H., Jansen T., Meyer B.T. (2024). Real-Time Multichannel Deep Speech Enhancement in Hearing Aids: Comparing Monaural and Binaural Processing in Complex Acoustic Scenarios. IEEE/ACM Trans. Audio Speech Lang. Process..

[9] Xiao T., Doclo S. Effect of Target Signals and Delays on Spatially Selective Active Noise Control for Open-Fitting Hearables. Proceedings of the 49th IEEE International Conference on Acoustics, Speech and Signal Processing (ICASSP).

[10] Dalga D., Doclo S. Combined Feedforward-Feedback Noise Reduction Schemes for Open-Fitting Hearing Aids. Proceedings of the IEEE Workshop on Applications of Signal Processing to Audio and Acoustics (WASPAA).

[11] Cox T.J., Barker J., Bailey W., Graetzer S., Akeroyd M.A., Culling J.F., Naylor G. Overview of the 2023 ICASSP SP Clarity Challenge: Speech Enhancement for Hearing Aids. Proceedings of the 48th IEEE International Conference on Acoustics, Speech and Signal Processing (ICASSP).

[12] Beit-On H., Lugasi M., Madmoni L., Menon A., Kumar A., Donley J., Tourbabin V., Rafaely B. Audio Signal Processing for Telepresence Based on Wearable Array in Noisy and Dynamic Scenes. Proceedings of the 47th IEEE International Conference on Acoustics, Speech and Signal Processing (ICASSP).

[13] Hafezi S., Moore A.H., Guiraud P., Naylor P.A., Donley J., Tourbabin V., Lunner T. Subspace Hybrid Beamforming for Head-Worn Microphone Arrays. Proceedings of the 48th IEEE International Conference on Acoustics, Speech and Signal Processing (ICASSP).

[14] Wang D., Chen J. (2018). Supervised Speech Separation Based on Deep Learning: An Overview. IEEE/ACM Trans. Audio Speech Lang. Process..

[15] Luo Y., Mesgarani N. (2019). Conv-TasNet: Surpassing Ideal Time–Frequency Magnitude Masking for Speech Separation. IEEE/ACM Trans. Audio Speech Lang. Process..

[16] Kolbæk M., Yu D., Tan Z.-H., Jensen J. (2017). Multitalker Speech Separation with Utterance-Level Permutation Invariant Training of Deep Recurrent Neural Networks. IEEE/ACM Trans. Audio Speech Lang. Process..

[17] Subakan C., Ravanelli M., Cornell S., Bronzi M., Zhong J. (2021). Attention Is All You Need in Speech Separation. IEEE Trans. Audio Speech Lang. Process..

[18] Guiraud P., Hafezi S., Naylor P.A., Moore A.H., Donley J., Tourbabin V., Lunner T. An Introduction to the Speech Enhancement for Augmented Reality (SPEAR) Challenge. Proceedings of the 17th International Workshop Acoustic Signal Enhancement (IWAENC).

[19] Erdogan H., Hershey J.R., Watanabe S., Le Roux J. Improved MVDR Beamforming Using Single-Channel Mask Prediction Networks. Proceedings of the Interspeech.

[20] Heymann J., Drude L., Haeb-Umbach R. Neural Network Based Spectral Mask Estimation for Acoustic Beamforming. Proceedings of the 41st IEEE International Conference on Acoustics, Speech and Signal Processing (ICASSP).

[21] Ochiai T., Delcroix M., Ikeshita R., Kinoshita K., Nakatani T., Araki S. Beam-TasNet: Time-Domain Audio Separation Network Meets Frequency-Domain Beamformer. Proceedings of the 45th IEEE International Conference on Acoustics, Speech and Signal Processing (ICASSP).

[22] Van Trees H.L. (2002). Optimum Array Processing: Part IV of Detection, Estimation, and Modulation Theory.

[23] Van Veen B.D., Buckley K.M. (1988). Beamforming: A Versatile Approach to Spatial Filtering. IEEE ASSP Mag..

[24] Capon J. (1969). High-Resolution Frequency-Wavenumber Spectrum Analysis. Proc. IEEE.

[25] Ehrenberg L., Gannot S., Leshem A., Zehavi E. Sensitivity Analysis of MVDR and MPDR Beamformers. Proceedings of the 26th IEEE Convention of Electrical and Electronics Engineers in Israel.

[26] Itzhak G., Cohen I. (2025). Robust Beamforming for Multispeaker Audio Conferencing Under DOA Uncertainty. IEEE/ACM Trans. Audio Speech Lang. Process..

[27] Itzhak G., Cohen I. Region-of-Interest Oriented Constant-Beamwidth Beamforming with Rectangular Arrays. Proceedings of the 2023 IEEE Workshop on Applications of Signal Processing to Audio and Acoustics (WASPAA).

[28] Konforti Y., Cohen I., Berdugo B. Array Geometry Optimization for Region-of-Interest Broadband Beamforming. Proceedings of the 17th International Workshop Acoustic Signal Enhancement (IWAENC).

[29] Itzhak G., Cohen I. (2025). STFT-Domain Least-Distortion Region-of-Interest Beamforming. IEEE Trans. Audio Speech Lang. Process..

[30] Frank A., Cohen I. (2025). Least-Distortion Maximum Gain Beamformer for Time-Domain Region-of-Interest Beamforming. IEEE Trans. Audio Speech Lang. Process..

[31] Braun S., Habets E.A.P. (2018). Linear prediction-based online dereverberation and noise reduction using alternating Kalman filters. IEEE/ACM Trans. Audio Speech Lang. Process..

[32] Yoshioka T., Nakatani T., Miyoshi M. (2009). Integrated speech enhancement method using noise suppression and dereverberation. IEEE Trans. Audio Speech Lang. Process..

[33] Nakatani T., Yoshioka T., Kinoshita K., Miyoshi M., Juang B.-H. Blind Speech Dereverberation with Multi-Channel Linear Prediction Based on Short Time Fourier Transform Representation. Proceedings of the 33rd IEEE International Conference on Acoustics, Speech and Signal Processing (ICASSP).

[34] Dietzen T., Spriet A., Tirry W., Doclo S., Moonen M., van Waterschoot T. (2018). Comparative analysis of generalized sidelobe cancellation and multi-channel linear prediction for speech dereverberation and noise reduction. IEEE/ACM Trans. Audio Speech Lang. Process..

[35] Boeddeker C., Nakatani T., Kinoshita K., Haeb-Umbach R. Jointly optimal dereverberation and beamforming. Proceedings of the 45th IEEE International Conference on Acoustics, Speech and Signal Processing (ICASSP).

[36] Nakatani T., Kinoshita K. (2019). A unified convolutional beamformer for simultaneous denoising and dereverberation. IEEE Signal Process. Lett..

[37] Yang W., Huang G., Brendel A., Chen J., Benesty J., Kellermann W., Cohen I. A bilinear framework for adaptive speech dereverberation combining beamforming and linear prediction. Proceedings of the 17th International Workshop Acoustic Signal Enhancement (IWAENC).

[38] Cohen I., Benesty J., Chen J. (2019). Differential Kronecker Product Beamforming. IEEE/ACM Trans. Audio Speech Lang. Process..

[39] Itzhak G., Cohen I. Differential and Constant-Beamwidth Beamforming with Uniform Rectangular Arrays. Proceedings of the 17th International Workshop Acoustic Signal Enhancement (IWAENC).

[40] Haykin S. (2002). Adaptive Filter Theory.

[41] Cioffi J., Kailath T. (1984). Fast, Recursive-Least-Squares Transversal Filters for Adaptive Filtering. IEEE Trans. Acoust. Speech Signal Process..

[42] Nemirovsky A., Itzhak G., Cohen I. A Bilinear Source Separation, Dereverberation, and Background Noise Suppression Algorithm for Augmented Reality Applications. Proceedings of the 50th IEEE International Conference on Acoustics, Speech and Signal Processing (ICASSP).

[43] Donley J., Tourbabin V., Lee J.-S., Broyles M., Jiang H., Shen J., Pantic M., Ithapu V.K., Mehra R. (2021). EasyCom: An Augmented Reality Dataset to Support Algorithms for Easy Communication in Noisy Environments. arXiv.

[44] Frost O.L. (1972). An Algorithm for Linearly Constrained Adaptive Array Processing. Proc. IEEE.

[45] Griffiths L.J., Jim C.W. (1982). An Alternative Approach to Linearly Constrained Adaptive Beamforming. IEEE Trans. Antennas Propag..

[46] Harville D.A. (1998). Matrix Algebra from a Statistician’s Perspective.

[47] Robusto C.C. (1957). The Cosine-Haversine Formula. Am. Math. Mon..

[48] Moore A.H., Hafezi S., Vos R.R., Naylor P.A., Brookes M. (2022). A Compact Noise Covariance Matrix Model for MVDR Beamforming. IEEE/ACM Trans. Audio Speech Lang. Process..

[49] Rix A.W., Beerends J.G., Hollier M.P., Hekstra A.P. Perceptual Evaluation of Speech Quality (PESQ)—A New Method for Speech Quality Assessment of Telephone Networks and Codecs. Proceedings of the 26th IEEE International Conference on Acoustics, Speech, and Signal Processing (ICASSP).

[50] Taal C.H., Hendriks R.C., Heusdens R., Jensen J. (2011). An Algorithm for Intelligibility Prediction of Time–Frequency Weighted Noisy Speech. IEEE Trans. Audio Speech Lang. Process..

[51] Le Roux J., Wisdom S., Erdogan H., Hershey J.R. SDR—Half-Baked or Well Done?. Proceedings of the 44th IEEE International Conference on Acoustics, Speech and Signal Processing (ICASSP).

[52] Hu Y., Loizou P.C. (2007). Evaluation of Objective Quality Measures for Speech Enhancement. IEEE Trans. Audio Speech Lang. Process..

[53] Hao X., Su X., Horaud R., Li X. FullSubNet: A Full-Band and Sub-Band Fusion Model for Real-Time Single-Channel Speech Enhancement. Proceedings of the 46th IEEE International Conference on Acoustics, Speech and Signal Processing (ICASSP).

[54] Lam M.W.Y., Wang J., Su D., Yu D. Mixup-Breakdown: A Consistency Training Method for Improving Generalization of Speech Separation Models. Proceedings of the 45th IEEE International Conference on Acoustics, Speech and Signal Processing (ICASSP).

[55] Braun S., Gamper H., Reddy C.K.A., Tashev I. Towards Efficient Models for Real-Time Deep Noise Suppression. Proceedings of the 46th IEEE International Conference on Acoustics, Speech and Signal Processing (ICASSP).

[56] Close G., Hain T., Goetze S. Hallucination in Perceptual Metric-Driven Speech Enhancement Networks. Proceedings of the 32nd European Signal Processing Conference (EUSIPCO).

